# An Account of a Recent Epidemic Dysentery, *of Alabama*

**Published:** 1839-07-13

**Authors:** H. V. Wooten

**Affiliations:** Lowndesboro’, Alabama


### MEDICAL EXAMINER.

DEVOTED TO MEDICINE, SURGERY, AND THE COLLATERAL SCIENCES.

No. 28.] PHILADELPHIA, SATURDAY, JULY 13, 1839. [Vol. II.

### AN ACCOUNT OF A RECENT EPIDEMIC DYSENTERY, of South Alabama. By H. V. Wooten, M. D., of Lowndesboro', Alabama.

It is not my purpose to offer any observations, having claim to novelty, on either the nature or treatment of dysentery. But, having recently enjoyed pretty extensive opportunities of witnessing its varying character, and of reducing to practice several of the plans of treatment which have been much spoken of, of late, I have thought proper to mention the results, with whatever remarks they may suggest.

Dysentery made its appearance in this place about the middle of April last, and continued, with more or less violence, until the middle of June. The town is situated upon a high ridge of land, which separates the low lands of the Alabama river from the large prairies. The soil is of a gravelly, or sandy character, with abundant water springs, and of the clearest freestone. This ridge is generally quite healthful, perhaps as exempt from autumnal bilious fevers as any spot in South Alabama, and it is rarely troubled with epidemics. This spring, however, dysentery has prevailed with considerable violence here, while the flat lands and prairies have been nearly exempt; nor has it prevailed anywhere in this part of the state, to my knowledge, so generally and violently as here.*

Varying as it did, in its symptoms and intensity, to facilitate the history of the treatment, I will divide it into two classes, or grades:—The first grade, characterized by violent griping and straining, with small muco-sanguineous evacuations, very frequent at the onset of the disease; pain mostly of the spasmodic character in the umbilical region, recurring at very short intervals; very little soreness, on pressure, in the beginning; pulse full, strong, and frequent; oppression in the epigastrium; vomiting sometimes troublesome; febrile reaction high; sometimes slight delirium; countenance flushed; eyes yellowish; tongue covered with a thick, dry, dark-brown fur, with a deep furrow in the centre; edges red and pointed; in many cases the urine was suppressed during the violence of the disease. These cases, if not early met by thorough treatment, generally ran a tedious and painful course.

Second grade:—Frequent evacuations, attended with pain, more or less severe; discharges larger and more watery than in the first grade, and pain only on evacuation; pulse a little frequent, but otherwise natural; febrile reaction very slight; tongue generally clear of fur, moist, edges red. The symptoms, if neglected, generally increased in violence, and the case became very nearly like one of the first grade, in the second stage. These cases, like the others, if not treated in proper time, ran a tedious course, recovering very slowly. It will be observed that the most important difference in the two grades, was the great biliary disorder which existed at the onset in cases of the first grade, and the comparative want of it in the second grade, which appeared to be more particularly an inflammation of the mucous coat of the intestines. I have only attempted a description of the symptoms in the early stages; they were subsequently modified by various causes, which, however, should not be set down to the disease.

I had witnessed the disease in the marshy regions of Georgia, in the same latitude; and although the treatment was generally successful, it was not altogether satisfactory,—and when the disease was presented to me here, I felt somewhat at a loss for the proper course to pursue. My confidence was first directed towards *Hope's astringent mixture.* The mineral acids have long been recommended in this disease; but my confidence in this particular preparation was greatly increased by the short article on the subject by Dr. Meigs, published in the first volume of the Medical Examiner. Having, during my pupilage in Philadelphia, formed a very high estimate of the professional ability and moral integrity of Dr. M., I felt no hesitancy in relying upon his recommendation of the mixture, and, accordingly, used it on the first opportunity. At first I gave it as opportunity offered, not regarding the character or stage of the case; but often, when "six doses," and more, had been given, no perceptible benefit was produced. In some cases, however, it appeared to have some influence in lessening pain, while all the other symptoms continued unchanged; I discovered, that when called to a case, after purges of calomel or castor oil, or both, had been used, the mixture was more likely to produce immediate benefit, and effect a cure. Having given it a fair trial, and found it unavailing in many cases, it was discontinued for a time, during which, combinations of calomel and opium, ipecacuanha, castor oil, &c., were used; and, on the resumption of the mixture, it was found to produce the most happy effect. The cases of its application to which I have alluded, were of the first, or biliary grade; and I am convinced that but little confidence can be placed in the acid mixture for their cure, until the heavy congestion of the liver and the portal circulation is removed; and, for this purpose, I have found it necessary to excite the secretory action of the liver by calomel or blue mass, pretty freely exhibited, and purged off by castor oil.

* Since writing the above, I have learned that it prevailed at the same time in a few other places, and in some with considerable fatality.

For exciting this action, I have always found the alterative action of the acid mixture entirely too weak; but, after this action has been well established, I know of no medicine to perpetuate such action, and, at the same time, address directly the irritation of the bowels, in which I have greater confidence than in the mixture alluded to.

In the second class, or milder grade, the mixture was more generally applicable, requiring often only a simple purge, as of castor oil, to prepare the way for its best effects; yet, it was found, in all cases, even of the lightest grade, to act much better after such purge, than before; and, in many cases of this class, which had been neglected until the glandular functions had become much disordered, the most thorough alterative action of calomel was required, before the beneficial action of the mixture could be induced. In these cases, however, I sometimes found the system ready prepared, the patient having taken these medicines before calling my attention to the case, and under such circumstances, I resorted to the acid mixture immediately, with satisfactory results. Witnessing these facts, and drawing their deductions, I was forced to the conclusion, that a preparation was always important, and frequently necessary, for the good effects of the acidulated mixture, more particularly in the bilious form of the disease.

For this purpose, when pain was severe, and reaction high, I found great benefit in free venesection; this not being indicated, or already done, I gave

Hydrag. chlorid. mit. gr. x.
Pulv. ipecac. et opii. āā gr. xx.

To be administered every third hour until four doses had been taken, to be followed, in three hours, by castor oil, and laudanum, if required, to alleviate pain. In some cases, where the pain was not so violent, and the stomach not irritable, one grain of ipecacuanha was substituted for, or added to the Dover's powder. The ipecacuanha seemed more particularly applicable to the cases of children. In illustration of my plan of treatment, I will give here a short sketch of a case: Mr. L., æt. twenty-four, of bilio-nervous temperament, good constitution, and moral habits, was attacked seven days ago with the mild, or second grade of the disease; this being treated by anodynes alone, gradually assumed a more formidable character, and at the time of visit was marked by heavy glandular disorder, with violent mucous inflammation of the intestines, presenting all the worst symptoms of the first grade, with considerable exhaustion. He was purged, without permanent benefit; the acid mixture was then instituted, f℥ii. being given every fourth hour. At the administration of the seventh dose, no appreciable benefit was produced. Tongue dry and thickly coated, with a deep furrow and red edges, as above described; febrile reaction high; slight delirium; oppression in the epigastrium; anorexia, &c. The calomel and opium course was then applied, on which copious evacuations of vitiated secretions, bile, &c., appeared. These ceased after the action of the medicine, and the evacuations had the appearance of returning to their former condition, sero-sanguineous, frequent and painful,—when Hope's mixture was again brought into service with immediate benefit, healthy secretions returning gradually, and general condition improving. This case fairly represents the ordinary results of my practice during the prevalence of the disease. It may be said that the calomel made the cure, without the aid of the mixture; this, however, would be an unjust conclusion, as the secretions were rapidly falling back to their former depravity when the mixture was resumed, which undoubtedly perpetuated the action excited by the calomel. It was sometimes necessary to repeat the calomel and opium daily for two or three days. As a simple purgative, I have found nothing so good as the castor oil; I have known several respectable practitioners, who used the Epsom salts altogether as a purgative in this disease, and from their recommendation I have been induced to use it myself,—but from the results that I have met, I greatly prefer the castor oil. When the irritation is primarily and chiefly in the intestines, and the liver in a healthy state, the salts, acting as a depletant, may do much good; I have myself seen much benefit arise from its use in such cases. But in this climate, the long and excessive heat of summer, keeping up an over-action of the liver, places that organ in an almost exhausted condition, rendering it the weak point for the invasion of disease,—and whenever disorder, from any cause ensues, affecting the portal circulation, the liver is generally found incompetent to the performance of its functions, which is not unfrequently our greatest hindrance in the removal of the disease; this is particularly so in the epidemic dysenteries of our climate. The salts, by producing a depletory effect, may, for a time, suspend the necessity for the liver's action, but will not restore to it that action which is important to the recovery of health. Moreover, when we have given an alterative, destined particularly for the secretory function of the liver, it seems to me absurd to set up a directly counter-action upon the intestinal exhalents. Sometimes, it is said, the suspended action of the liver is owing to an engorgement of its vessels, and that the salts, by their depletory action, relieve this obstruction. Granting this fact, the salts should be administered, in such cases, before the calomel, or other alterative, and not, as is usually done, after it; but, where no other than depletory action is required, I prefer the lancet. Castor oil, on the contrary, producing no unnatural discharge from the bowels, but simply exciting the peristaltic motion of the intestines, thereby emptying them of their contents—perhaps the products of the alterative—seems to me an apt adjunct to the use of calomel, when purgation is desired. These considerations, with the recollection of the fact that mucous secretion, and not serous exhalation, is the natural product of the intestinal mucous membrane, have led me to the constant preference of the oil in this complaint, and all others where a promotion of hepatic action is desirable.

When the tenesmus and griping were severe, great benefit was frequently derived from a combination of rhubarb and Dover's powder, āā gr. x, given every second hour until the pain and discharges were moderated. It often quieted the irritation, and produced consistent evacuations, thus greatly relieving the worst symptoms of the disease. This was rarely permanent, however, unless the secretions had been previously restored to a proper state; consequently, I have confidence in this combination no further than its alleviation of irritation, for which it is an excellent remedy. The balsam copaiva was also tried; I did not resort to its use in the early part of the epidemic, nor until after I had become convinced of the propriety of first restoring the secretions in all serious cases, which, I am satisfied, was the proper time for its application. I observe in the essay of Dr. La Roche on the subject, that in all the cases which he instances of its best effects, it was given after purgation; and it seems that those cases in which the blue mass had been previously used, were the most signally benefited by the balsam copaiva. The cases which he reports were prepared by other medicines for the action of the copaiva, or such as had become chronic, the inflammation of the bowels being the most important disease to be remedied. In either case, its use corresponds with my experience given under these circumstances,—its remedial powers were strikingly happy. In one case, that of a child, in which every other remedy had failed, and ulceration had apparently ensued, it acted promptly and efficiently, producing a speedy recovery. In some cases I have no doubt, that, from long neglect, and the violence of inflammation, it may become so permanent, and the discharge from the mucous membrane so habitual, as greatly to counteract the action of alteratives on the liver and other secretory organs, in which circumstances it becomes necessary to address our remedies to the mucous membrane first. And, further, I have no doubt that this state of things occurs in a greater proportion of cases in the practice of Dr. La Roche and Dr. Meigs, than mine, or the general practice in this climate, not owing particularly to the causes just mentioned, but to the great difference of climate, or rather its effects on the human system; for in nearly all these disorders in this climate, the liver is primarily involved, while such is not the case in Philadelphia, which may well account for the different results from the use of the same remedies. In cases where mucous inflammation was to be particularly addressed, I found the balsam copaiva a very excellent remedy. I use it according to Eberle's formula:

℞. Balsam copaiv. ℥ss.

Pulv. gum arab. ʒii.

Sacch. albi. ʒiii.

Aquæ. font. ℥viii.

Tinct. Opii ʒi.

A tablespoonful every fourth hour.

In the chronic stages of mucous inflammation, where the existence or approach of ulceration is apprehended, I would rely more on the copaiva than any other medicine.

The acetate of lead was used in several cases, but its use was so barren of good as to cause its early abandonment. Opiates, alone, were used in some cases, but they only had a temporary anodyne effect, the disease always returning with its former violence on the withdrawal of the medicine. The use of anodyne and mucilaginous injections was resorted to in many cases; but, unfortunately, those cases which most urgently demanded their influence, could not tolerate their presence, and they were immediately rejected, so that benefit from this source was rarely to be obtained.

In a very few cases, which appeared rather tedious under the ordinary remedies, I tried the use of nux vomica, as recommended by Mr. Vaux, through Dr. Armstrong. It seemed to exercise some anodyne influence; but, on increasing the dose to the amount advised, (seven grains,) its peculiar constitutional effects were such as to stop its use. I did not make a full trial of this article.

In many cases where griping was frequent and severe, much benefit was derived from the warm bath.

I have observed the disease closely, not only here, but in the same latitude elsewhere, as above mentioned, and in the practice of an excellent physician. Never being wholly satisfied with any particular remedy, or course of treatment, and always viewing the disease as one distressing in its operation, and dangerous in its results, I have, while watching its varying character, been equally watchful in applying various remedies, as I thought them indicated. The comparative efficiency of these I have thought it unnecessary to mention, except, as applied during the *same epidemic*, frequently in the *same case, and altogether under the same circumstances*,—and this, the trials being pretty extensive, I have thought interesting;—on which account I have given the above sketch, from which the following conclusions may be drawn:

1st. That, in all cases of dysentery, occurring in this climate, the liver is either primarily or secondarily involved, producing a suspension of its secretory action, which has a direct tendency to increase and perpetuate the phlogosis of the bowels, and that this organ must be restored to its due action before a cure can be effected.

2d. That, to restore the secretory action of the liver, (depletion being premised, when indicated,) nothing can be fully relied on but mercurial preparations, given in quantity and continuation sufficient to the purpose; and that calomel is to be preferred, succeeded by castor oil, to produce gentle, but free purgation.

3d. That, to alleviate pain, and stay the spasmodic action of the intestines, opiates should be given with the calomel, in the quantity required, and, the stomach allowing, ipecacuanha should be added, with a view to its diaphoretic effect.

4th. That, when the secretory action of the organs involved has been restored, and it is desirable to perpetuate that action, and at the same time allay mucous irritation, nothing is more to be relied on than Hope's nitrous acid mixture, freely given.

5th. That, where the advanced stage of mucous inflammation is to be combated, as the main object in view, the balsam copaiva is the best remedy.

6th. That, much suffering may be saved by the proper use of the warm bath, and anodyne injections, when they can be retained.

7th. That, under all circumstances, a regular action of the liver, and other secretory organs, should never be lost sight of.

8th. That proper dietetic treatment is all-important.

It may be proper, in conclusion, to remark, that I am indebted to my friend and copartner, Dr. H. P. Perry, for much of the opportunity which I have had of observing the disease, and the use of remedies; and further, that during the entire prevalence of the disease, we had but two cases to terminate fatally, both children under two years old, and one suddenly, evidently from intussusception.

*Lowndesboro'*, June 21st, 1839.

