# Erratum in No. 28

**Published:** 1839-08-10

**Authors:** 


Erratum in No. 28.—In Dr. Wooten's paper on Dysentery, page 438, line 35, for "Pulv. ipecac. et opii $\overline{aa}$ gr. xx.," read *Pulv. ipecac. et opii* gr. x. *ad* xx.

